# Evaluation of Prostate-Specific Membrane Antigen (PSMA) Immunohistochemical Expression in Early-Stage Breast Cancer Subtypes

**DOI:** 10.3390/ijms25126519

**Published:** 2024-06-13

**Authors:** Natalia Andryszak, Paweł Kurzawa, Monika Krzyżaniak, Michał Nowicki, Marek Ruchała, Dariusz Iżycki, Rafał Czepczyński

**Affiliations:** 1 Department of Endocrinology, Metabolism and Internal Medicine, Poznan University of Medical Sciences, 60-355 Poznan, Polandczepczynski@ump.edu.pl (R.C.); 2 Department of Oncological Pathology, Hospital of Lord’s Transfiguration, Poznan University of Medical Sciences, 60-101 Poznan, Polandmonika.krzyzaniak@usk.poznan.pl (M.K.); 3 Department of Histology and Embryology, Poznan University of Medical Sciences, 60-781 Poznan, Poland; 4 Department of Cancer Immunology, Poznan University of Medical Sciences, 60-101 Poznan, Poland; dizycki@gmail.com

**Keywords:** breast cancer, PSMA, prostate-specific membrane antigen

## Abstract

Breast cancer, known for its diverse subtypes, ranks as one of the leading causes of cancer-related deaths. Prostate-specific membrane antigen (PSMA), primarily associated with prostate cancer, has also been identified in breast cancer, though its role remains unclear. This study aimed to evaluate PSMA expression across different subtypes of early-stage breast cancer and investigate its correlation with clinicopathological factors. This retrospective study included 98 breast cancer cases. PSMA expression was examined in both tumor cells and tumor-associated blood vessels. The analysis revealed PSMA expression in tumor-associated blood vessels in 88 cases and in tumor cells in 75 cases. Ki67 expression correlated positively with PSMA expression in blood vessels (*p* < 0.0001, RSpearman 0.42) and tumor cells (*p* = 0.010, RSpearman 0.26). The estrogen and progesterone receptor expression correlated negatively with PSMA levels in blood vessels (*p* = 0.0053, R Spearman −0.26 and *p* = 0.00026, R Spearman −0.347, respectively). Human epidermal growth factor receptor 2 (HER2) status did not significantly impact PSMA expression. We did not detect any statistically significant differences between breast cancer subtypes. These findings provide evidence for a heterogenous PSMA expression in breast cancer tissue and suggest its correlation with tumor aggressiveness. Despite the limited sample size, the study provides valuable insights into the potential of PSMA as a prognostic, diagnostic, and therapeutic target in the management of breast cancer.

## 1. Introduction

Breast cancer is the most common cancer in women, as reported by the American Cancer Society. It also ranks as the second leading cause of cancer-related deaths among women, second only to lung cancer [1]. In the diagnosis and management of breast cancer, it is crucial that we determine the histological type of the tumor and identify the biological subtype through an immunohistochemical evaluation. This classification relies on assessing the expression of steroid receptors, such as estrogen and progesterone receptors, as well as the HER2 status and the Ki-67 proliferation index. The identification of four main biological subtypes, namely, luminal A-like, luminal B-like, HER2-positive, and triple-negative breast cancer (TNBC), forms the basis for selecting appropriate systemic treatment strategies [2].

Prostate-specific membrane antigen (PSMA) is a transmembrane glycoprotein primarily known for its overexpression in prostate cancer cells. While PSMA is physiologically expressed in various normal tissues, including the prostate epithelium, small intestine, renal tubules, and salivary glands, its expression is significantly higher (100 to 1000 times) in prostate cancer cells, correlating with tumor aggressiveness [3,4].

The precise physiological role of PSMA is not fully understood, but it is predominantly associated with enzymatic peptidase activity, involved in folate and glutamate metabolism, as well as signaling pathway activation [5]. As early as in 1999, Chang et al. demonstrated the presence of PSMA in different neoplastic cell types, including breast cancer, kidney cancer, bladder cancer, and melanoma [6]. PSMA expression has been detected in the neovasculature of various tumor types, including breast, renal, colon, and transitional cell carcinomas, where it plays a role in regulating the invasion of angiogenic endothelial cells. Since then, numerous studies have highlighted the involvement of PSMA in neoangiogenic processes within cancer cells [5,7,8]. Conway et al. indicated that PSMA regulates angiogenesis by directly inducing the LQ dipeptide, which promotes endothelial adhesion and invasion. These findings suggest that the inhibition of PSMA could potentially lead to the development of new angiogenic therapies [9].

The PSMA has been detected in breast cancer cells, and, to this day, there have been few studies evaluating the PSMA expression in breast cancer tumors as a promising new therapeutic target [10,11,12]. Wernicke et al. proved the existence of PSMA expression in the tumor-associated vasculature of primary breast cancer and brain metastases in 92 cases [13]. They indicated that normal breast tissue and cancer cells were PSMA-negative, whereas Kasoha et al. studied PSMA expression in primary breast cancer and metastases and indicated a significantly higher expression of PSMA in metastatic lesions, in both tumor-associated vessels and cancer cells [14]. However, they did not find differences between breast cancer subtypes and PSMA expression.

PSMA, being an integral membrane cell surface protein, serves as an excellent target for radionuclide ligands, which have been successfully utilized in medical imaging. Positron emission tomography (PET) examinations, based on PSMA ligands labeled with ^68^Ga or ^18^F, highly effective nuclear imaging tracers, enable the analysis of cancer cell distribution in prostate cancer. Hence, PET/CT using ^68^Ga-labeled PSMA ligands, due to its high sensitivity, has become a routine tool for staging and detecting recurrence in prostate cancer. PSMA overexpression is an attractive molecular target not only for imaging but also for radionuclide therapy, that has recently been introduced in prostate cancer with promising results [15].

We hypothesize that prostate-specific membrane antigen (PSMA) is present in breast cancer tissue and that its expression levels are correlated with the aggressiveness of different subtypes of the disease. Specifically, we anticipate that higher levels of PSMA expression will be associated with more aggressive breast cancer subtypes, indicating its potential role as a biomarker for disease progression and aggressiveness.

Understanding the variation in PSMA expression across breast cancer subtypes is crucial for tailoring treatment approaches and potentially identifying subtype-specific therapeutic targets. The aim of our study was to evaluate the PSMA expression in younger patients (≤50 years old) with early-stage breast cancer and assess its distribution across breast cancer subtypes. Additionally, we have investigated the correlation of immunohistochemical factors related to breast cancer with PSMA expression.

## 2. Results

In our study, 98 breast cancer patients were included, with grade 3 cancer, with a mean age of 41.1 ± 8.2 years, and with the following distribution among the subtypes (Table 1):

- HER2-positive: 29 cases, including 12 HER2 non-luminal and 17 HER2 luminal cancers;
- Triple-negative breast cancer (TNBC): 19 cases;
- Luminal A-like: 25 cases;
- Luminal B-like: 25 cases.

PSMA expression was confirmed in the tumor blood vessels in 88 out of 98 cases, and in the tumor cells in 75 cases. The overexpression of PSMA was predominantly observed in the blood vessels (42 out of 88 cases), while, within the tumor, a focal expression was seen in the majority of cases (59 out of 75 cases) (Table 2 and Table 3).

We analyzed the differences in PSMA expression between cancer subtypes. No significant difference in PSMA expression was observed in the blood vessels among different breast cancer subtypes (luminal, HER2, and TNBC); however, it approached statistical significance (*p* = 0.061). Notably, PSMA expression was present in the blood vessels of all TNBC cases, and the highest level of PSMA expression was also observed in the TNBC subtype. There was no significant difference in PSMA expression in tumors between subtypes either (*p* = 0.239). The strongly positive expression of PSMA in tumor cells was observed only in the TNBC subtype. The luminal A-like subtype showed the lowest expression of PSMA in the tumor.

We analyzed the correlations between PSMA expression levels and various immunohistochemical factors of breast cancer subtypes.

### 2.1. Ki67 Level

**Figure 1:**
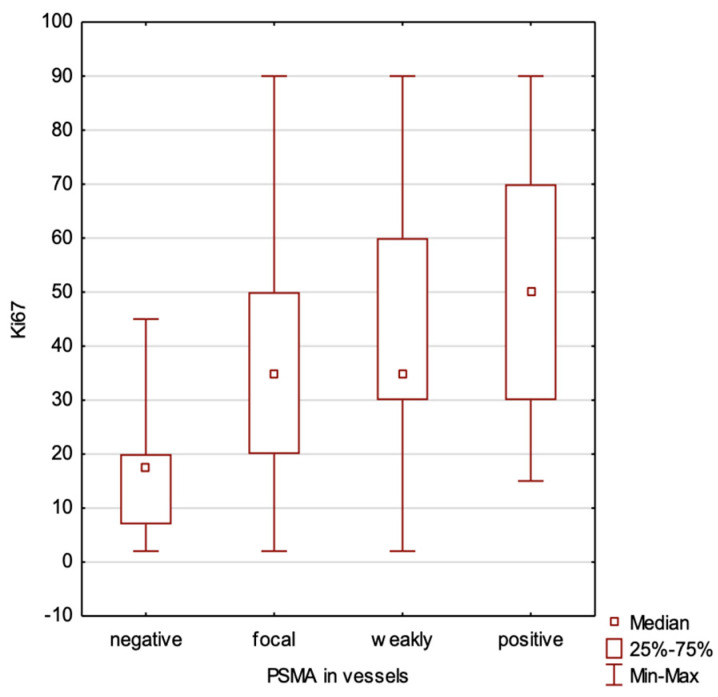
Correlation between Ki67 level and PSMA expression in blood vessels of the tumor.

A higher Ki67 expression was correlated with an increased PSMA expression in both the blood vessels (*p* < 0.0001, R Spearman 0.42) and within the tumor (*p* = 0.010, R Spearman 0.26) (Figure 1).

### 2.2. Estrogen Receptor Expression

**Figure 2:**
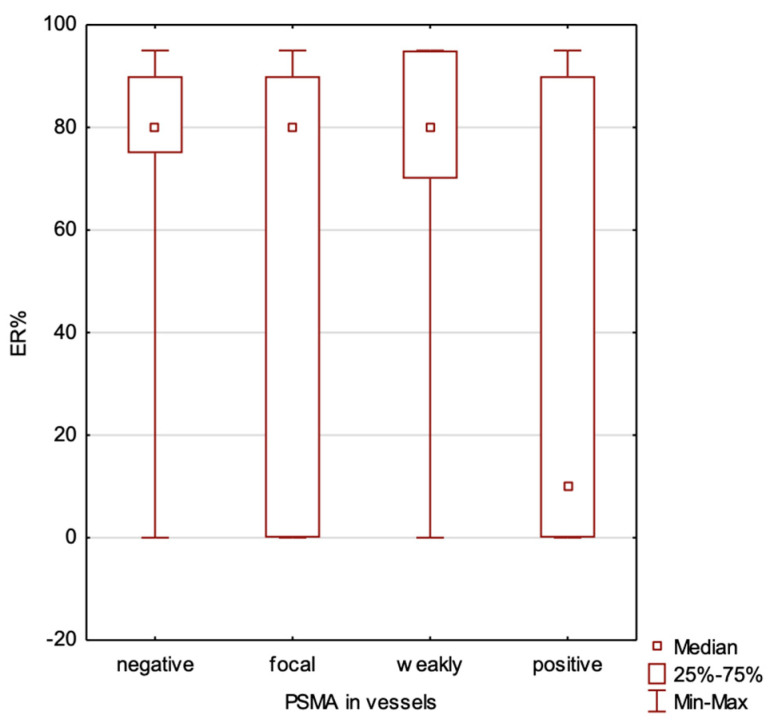
Correlation between estrogen receptor percentage level and PSMA expression in blood vessels of the tumor.

We compared cases with positive vs. negative estrogen receptor expression. Tumors with no estrogen receptor expression exhibited significantly higher levels of PSMA expression both in the blood vessels (*p* = 0.024) and within the tumor (*p* = 0.011). We have found a significant negative correlation between the percentage of estrogen receptor expression and PSMA expression in the blood vessels (*p* = 0.0053, R Spearman (−0.26)) but not in the tumor (*p* = 0.053) (Figure 2).

### 2.3. Progesterone Receptor Expression

**Figure 3:**
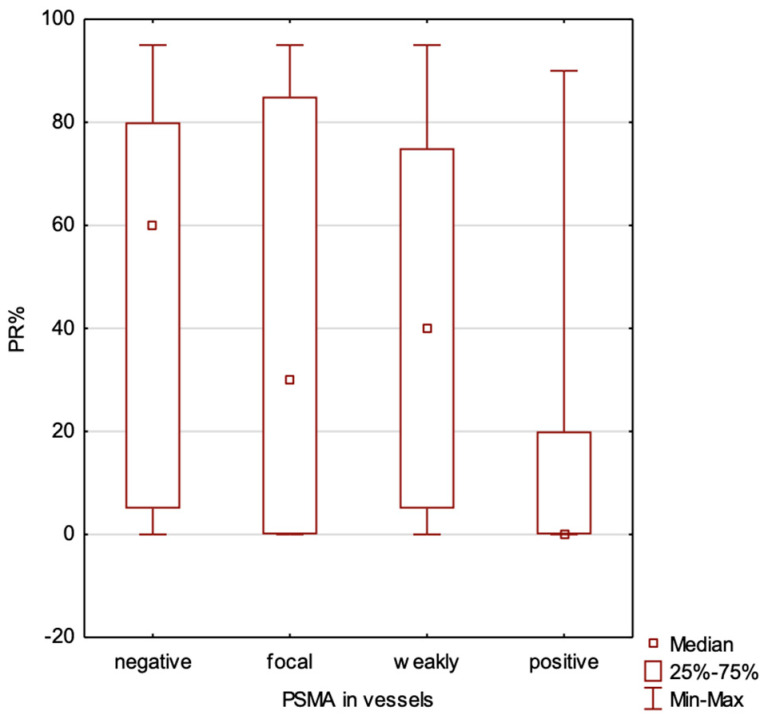
Correlation between progesterone receptor percentage level and PSMA expression in blood vessels of the tumor.

We compared cases with a positive vs. negative progesterone receptor expression. The absence of progesterone receptor expression was associated with higher levels of PSMA expression in the blood vessels (*p* = 0.0046), while no significant differences were observed in PSMA expression within the tumor (*p* = 0.0996). Our analysis has indicated a significant negative correlation between the percentage of progesterone receptors and PSMA expression in the blood vessel (*p* = 0.00026, R Spearman (−0.347)), but not in the tumor (*p* = 0.166) (Figure 3).

### 2.4. HER2 Status

There were no significant differences between cases with a positive or negative HER2 status in either the blood vessels (*p* = 0.8889) or within the tumor (*p* = 0.932).

## 3. Discussion

Prostate-specific membrane antigen is a transmembrane glycoprotein which plays various roles depending on the localization and physiological status of tissue. In the nervous system, PSMA plays a crucial role as glutamate carboxypeptidase II (GCP II), also known as NAALADase, in hydrolyzing neuronal dipeptide N-acetylaspartylglutamate (NAAG). A dysregulation of the glutamatergic neurotransmission by NAAG is implicated in schizophrenia, seizure disorders, Alzheimer’s disease, Huntington’s disease, and amyotrophic lateral sclerosis [4]. The exact physiological role of PSMA in prostate epithelial cells remains unknown. However, it is well-established that PSMA is overexpressed in prostate cancer cells and is utilized as a tumor marker and imaging target in PET [3]. In prostate cancer, PSMA overexpression has been linked to various processes, including cell migration, invasiveness, folate uptake, bone metastases, and an unfavorable prognosis [7]. The study by Evans et al. has shown that reducing PSMA expression led to cell cycle arrest, decreased cell proliferation, and diminished invasiveness in prostate cancer, which makes PSMA a tool for prostate cancer treatment [16]. The overexpression of PSMA in prostate cancer has been utilized in treatment using a PSMA ligand radiolabeled with beta- or alpha-particle-emitting nuclides such as lutetium-177 (177Lu) or actinium-225 (225Ac) [17]. An analysis of the effectiveness of the radionuclide therapy using ^177^Lu-PSMA-617 in nearly 150 patients with prostate cancer demonstrated high response rates and safety. In addition, its combination with androgen receptor was effective in 165 patients with metastatic castration-resistant prostate cancer [17,18]. Based on the results of a multicenter trial on 831 patients, the treatment has been recently registered in a number of countries [15]. With the notable success of PSMA radioligand therapy in treating prostate cancer patients, there is an emerging question regarding the potential benefits of applying it to other cancers that express PSMA either on the tumor cells themselves or on the tumor-associated neovasculature.

Many studies have revealed the expression of PSMA in various cancers besides prostate cancer. ^68^Ga-PSMA-11 uptake in PET/CT scans has been reported, for example, in glioma [19], melanoma, lung cancer, breast cancer [10,11,12,13], renal carcinoma, colorectal cancer, and gastric cancer [8]. The accumulation of ^68^Ga-PSMA-11 in breast cancer tissue has been demonstrated in a few studies [20]. The successful identification of breast cancer lesions by radiolabeled PSMA ligand shows that the PSMA molecule is a potential target for diagnosis, and also for potential radionuclide therapy in this malignancy [21].

The prognosis and management of breast cancer are known to vary based on its immunohistochemical subtypes. Molecular and genetic studies contributed to a better understanding of breast tumor pathophysiology and demonstrated its heterogeneity [22]. The diverse clinical, pathological, and therapeutic response profiles exhibited by different subtypes of breast cancer have a significant implication for treatment strategies aimed at the optimization of the outcome. TNBC is considered the most aggressive subtype with a high metastatic potential and the lowest survival rate. Due to the absence of estrogen and progesterone receptors, high Ki67 expression, and negative HER2 status, systemic treatment for TNBC primarily relies on chemotherapy. Targeted therapy poses challenges in TNBC patients due to its significant heterogeneity [23].

Several studies have demonstrated that PSMA expression is associated with the invasiveness of the prostate cancer cells. The PSMA’s transmembrane localization is proven to have a critical role in the angiogenesis and generation of metastases [4,24]. Conway et al. indicated that PSMA regulates integrin activation and signal transduction in a laminin-specific manner to direct angiogenic endothelial cell adhesion and invasion. These findings suggest that the overexpression of PSMA could potentially lead to the development of new angiogenic therapies, by suppressing PSMA [9].

**Figure 4:**
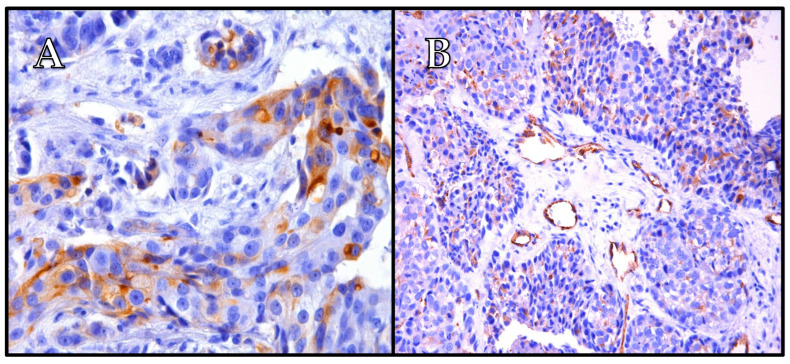
The case of one patient with TNBC, Ki67 85%, grade 3. Immunohistochemistry revealed strongly positive expression of PSMA in tumor cells (cytoplasm + membrane) (**A**) and positive expression of PSMA in tumor-associated vessels (**B**). The patient was diagnosed at stage IIA. (**A**) Mag. ×400; and (**B**) mag. ×200.

**Figure 5:**
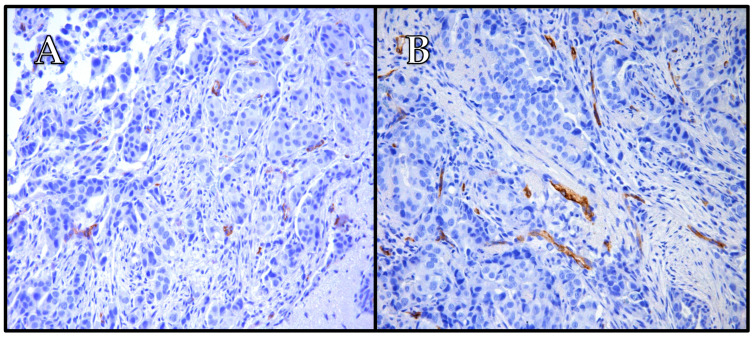
The case of one patient with HER2-luminal breast cancer, 90% estrogen receptor expression, 2% progesterone receptor expression, Ki67 45%, grade 2. Immunohistochemistry revealed focal positive expression of PSMA in tumor cells (**A**) and positive expression of PSMA in tumor-associated vessels (**B**). The patient was diagnosed at stage IIA. (**A**) Mag. ×200; and (**B**) mag. ×200.

**Figure 6:**
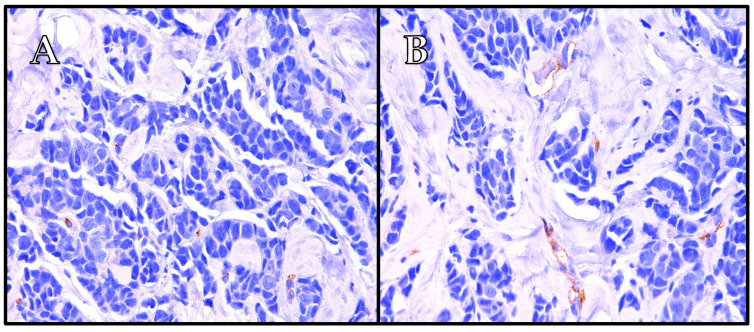
The case of one patient with luminal A-like breast cancer, 90% estrogen receptor expression, 70% progesterone receptor expression, Ki67 2%, grade 2. Immunohistochemistry revealed negative expression of PSMA in tumor cells (**A**) and weakly positive expression of PSMA in tumor-associated vessels (**B**). The patient was diagnosed at stage IB. (**A**) Mag. ×400, and (**B**) mag. ×400.

The findings of our study indicate that PSMA expression in breast cancer is predominantly observed in the blood vessels, with varying levels across different subtypes (Table 2 and Table 3, Figure 4, Figure 5 and Figure 6). Its vascular localization supports the hypothesis that PSMA may play a role in angiogenesis in breast cancer, similarly to that in prostate cancer. We observed a negative correlation between PSMA expression in the blood vessels and the expression of estrogen and progesterone receptors (Figure 2 and Figure 3). The luminal subtypes of breast cancer, which are characterized by the presence of steroid receptors, generally have better prognoses and lower proliferation rates. Furthermore, we found that higher levels of Ki67 expression were associated with increased PSMA expression in both the blood vessels and within the tumor (Figure 1), suggesting that more aggressive tumors exhibit higher levels of PSMA expression (Figure 4). Interestingly, the HER2 status did not significantly affect the PSMA expression in our study. These results provide valuable insights into the expression patterns of PSMA in breast cancer and have potential implications for subtype-specific diagnosis and treatment strategies. The strong correlation between PSMA expression, a higher proliferation, and metastases suggests its potential as a prognostic and therapeutic target in breast cancer, especially in the most aggressive subtype—TNBC.

Our study presents significant clinical implications, particularly in the context of patients with TNBC, a subtype often characterized by chemotherapy resistance and rapid disease recurrence after treatment. The overexpression of PSMA may open up additional imaging opportunities, using PET/CT. Patients with advanced-stage cancer may potentially benefit from targeted radioligand therapy, as it can selectively destroy the vessels supplying the tumor microenvironment, overcome tumor resistance, and spare normal tissues that do not express PSMA. Furthermore, the high PSMA expression might be harnessed in the development of novel immunotherapy approaches, aiming to inhibit angiogenesis. As reported by Morgenroth et al., the homogenous intratumoral tracer distribution results from the endothelial and epithelial expression of PSMA [25]. This expression pattern allows the simultaneous targeting of both tumor tissue compartments, the neovasculature and epithelial cancer cells. As PSMA expression is specific for to tumor-associated neovasculature (but not for normal endothelium), PSMA seems an optimal target for the targeted radionuclide therapy of TNBC in contrast to general angiogenetic targets as integrins or VEGF.

Other authors have found that binding a radiolabeled ligand to PSMA to the endothelial and malignant cells induces an efficient ligand internalization [26,27]. This allows intracellular drug delivery and trapping, which increases the intracellular dose accumulation. As demonstrated in vivo, lutetium-177-labeled PSMA ligands induce double-strand DNA breaks that are responsible for the cytotoxic effect of radionuclide therapy [28]. A recently published prospective study explored the potential of prostate-specific membrane antigen (PSMA) as a diagnostic and therapeutic target in TNBC, performing [^18^F]PSMA-1007 PET/CT and comparing it head-to-head with the standard-of-care [^18^F]FDG PET/CT in 10 patients. The study revealed a comparable uptake of [^18^F]PSMA-1007 and [^18^F]FDG in primary and metastatic lesions of triple-negative breast cancer, which underscores the clinical potential of PSMA [29].

Overall, our study has certain limitations. Despite a substantial number of patients included in the study, the sample size has not been large enough to detect statistically significant differences between breast cancer subtypes. Furthermore, the clinical data correlation was not assessed in this study. Future investigations should aim to correlate PSMA expression with clinical outcomes such as overall survival (OS) and progression-free survival (PFS). To introduce PSMA as a new imaging target, further research is needed to evaluate PET/CT with the PSMA radioligand as a novel imaging modality in patients with breast cancer. Furthermore, it is essential that we assess the effectiveness of targeted radioligand therapy in breast cancer patients. This is a single-center study. Biopsy material was used in order to maintain consistency. It must be pointed out, however, that biopsy specimens pose another limitation as they may not be as representative of the entire tumor as slides obtained surgically.

## 4. Materials and Methods

### 4.1. Population

This retrospective study received approval from the Institutional Review Board of Poznan University of Medical Sciences.

The inclusion criteria for the study comprised:

- Diagnosis of stage I or II breast cancer, indicating early-stage disease;
- Age lower than 50 years at the time of diagnosis.

Exclusion criteria included

- History of another malignancy;
- History of any prior oncological treatment;
- History of autoimmune disease;
- Undergoing immunosuppressive therapy.

Immunohistochemistry data, including the expression of estrogen and progesterone receptors, HER2 status, and Ki67 level, were analyzed in the tumor samples.

**Figure 7:**
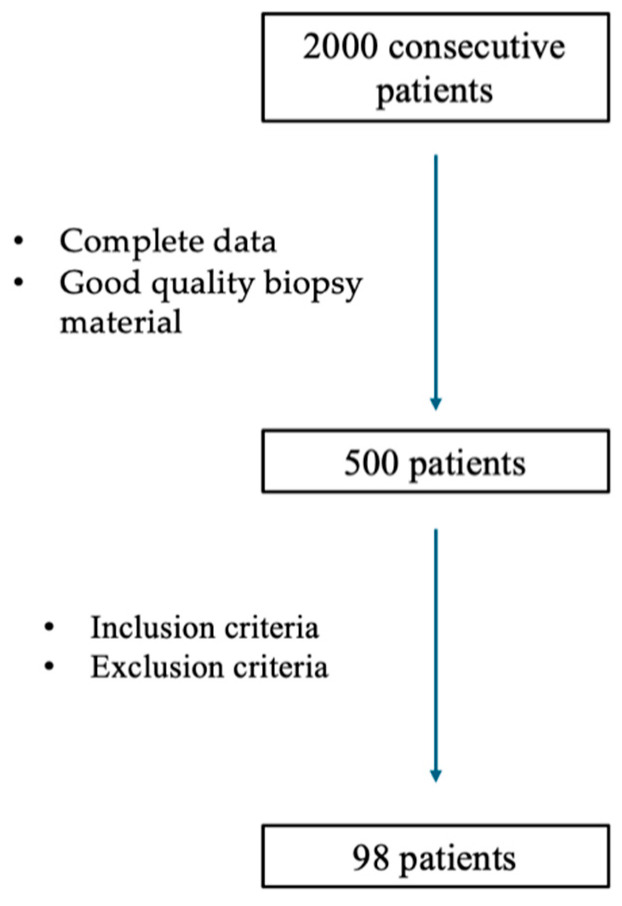
Flowchart of selecting patients for the study.

The study involved the analysis of data from over 2000 consecutive patients with breast cancer who were diagnosed in the Oncology Department between January 2017 and December 2022. Among them, 500 patients had complete data and good-quality paraffin sections available for analysis. After applying the predefined inclusion criteria, a total of 98 patients fulfilled the criteria and were included in the study (Figure 7).

The age range of the participants was between 32 and 50 years old, with a mean age of 41.1 years old. The analysis focused on formalin-fixed, paraffin-embedded tissue blocks obtained from core biopsy specimens collected from each of the patients before treatment, specifically at the time of diagnosis. Detailed clinicopathological data were collected from pathology and clinical records.

### 4.2. Immunohistochemistry

For immunohistochemical PSMA expression in study group, 98 slides were performed. They contained tissue samples from 98 patients. Each slide contained specimen from one patient and external control with four different normal tissue cores: tonsil, appendix, liver, and pancreas. The samples were obtained from biopsies to maintain consistency and ensure that all specimens were collected before the initiation of treatment. This approach was adopted to account for the varied treatment paths, with some patients undergoing immediate surgery and others receiving neoadjuvant chemotherapy.

Serial 4-micrometer tissue cut sections obtained from the paraffin block containing breast cancer specimens were applied to special immunohistochemistry-coated slides with external control cores cut before. Then, slides were baked for minimum of 60 min in 60 °C. A prostate-specific membrane antigen, clone 3E6, a rabbit recombinant monoclonal FOLH1 antibody (ab133579), clone number EPR6253, was used to demonstrate expression of this membrane glycoprotein. Immunohistochemical staining was performed at a working dilution of 1 in 300. FOLH1, or folate hydrolase 1, and PSMA are encoded by the same gene and represent the same protein, responsible for different pathways depending on its localization.

Staining was performed on a fully automated immunohistochemistry slide stainer Autostainer Link 48 (Agilent Technologies (Santa Clara, CA, USA)). Staining protocol parameters were based on heat-induced epitope retrieval (HIER) using FLEX TRS Low (K8005). In a pretreatment step, the heating time of 20 min at 97 °C was applied. Incubation of the primary antibody PSMA (IR089) was 30 min long. EnVision FLEX (K8004) was used as detection system.

The antigen was localized using Chromogen DAB-3.3 applied in all preparations. Slides were then stained with Hematoxylin (K8008) for 5 min. After staining, the sections were dehydrated, cleared, and mounted using permanent mounting method.

Each slide contained specimen from one patient and external control, which was used in Pathology Department for daily quality control purpose. The external control contained four different normal tissue cores: tonsil, appendix, liver, and pancreas. Daily quality control helped to exclude any false-positive staining reactions and verify used detection system for “nonspecific” (unwanted) binding of the components. As an internal positive control, the prostate tissue was used to evaluate antibody specificity and sensitivity.

The PSMA expression was evaluated and categorized into four categories: negative, focal, weakly positive, and strongly positive. This assessment was performed separately for tumor cells and tumor vessels. In our study, the positive expression in tumor cells was assessed if it occurred in the cell membrane, cytoplasm, or both membrane and cytoplasm. The classification of stained vessels’ expression levels relied on evaluating positive expression specifically within the endothelial cells. The evaluation of PSMA reactivity in tumor cells and tumor vessels was conducted in a blinded manner, without knowledge of the clinical data.

Microscopic slide images were taken using Leica microscope and Leica camera with 20× and 40× objectives.

### 4.3. Statistical Analysis

The calculations were performed using the Statistica (v.13, TIBCO, Palo Alto, CA, USA) and PQStat (v.1.8.6, PQStat Software, Poznan, Poland) software. Sample size calculation was performed to justify the number of participants included in the study. A significance level of α = 0.05 was adopted. A result was considered statistically significant when *p* < α. The normality of variable distributions was assessed using the Shapiro–Wilk test. Due to non-normal distribution, the Kruskal–Wallis test with the Dunn–Bonferroni multiple comparisons test was used to compare variables among multiple groups. The Jonckheere–Terpstra test was used to determine if there was a significant trend. The Spearman’s rank correlation coefficient (Rs) was calculated to examine the relationships between variables. The Fisher–Freeman–Halton test was used to analyze the associations between categorical variables.

## 5. Conclusions

This study represents a pioneering effort in assessing PSMA expression and its correlation among subtypes of early-stage breast cancer, with a specific focus on younger patients. Our findings solidify the presence of PSMA expression within breast cancer cells, notably within tumor-associated blood vessels. Importantly, our analysis unveils a noteworthy association between heightened PSMA expression and the aggressiveness of breast cancer cells, hinting at its potential role in angiogenesis and metastatic dissemination.

These discoveries not only contribute to our understanding of breast cancer pathology but also hold promising implications for future diagnostic and therapeutic endeavors in younger patient cohorts. The identification of PSMA’s involvement in breast cancer progression underscores the urgency of exploring novel treatment modalities, particularly for aggressive subtypes prevalent in this demographic.

Of particular interest is the overexpression of PSMA in triple-negative breast cancer, a highly aggressive subtype with limited treatment options. Harnessing this knowledge could pave the way for innovative therapeutic strategies tailored to combat the unique challenges posed by this subtype.

In essence, our study lays a crucial foundation for further research aimed at unraveling the intricacies of PSMA-mediated mechanisms in breast cancer pathogenesis. By shedding light on the potential of PSMA as a diagnostic marker and therapeutic target, we inch closer to personalized treatment approaches that hold the promise of improved outcomes for younger breast cancer patients.

## Figures and Tables

**Table 1:** Overview of the breast cancer patient cohort in the study—subtypes and TNM stage. All patients were diagnosed with grade 3 cancer.

Breast Cancer Subtype	Number of Patients		I Stage	II Stage
HER2-positive	29	12 non-luminal	6	6
		17 luminal	9	8
Triple-negative	19		5	14
Luminal A	25		19	6
Luminal B	25		17	8

**Table 2:** Distribution of estrogen receptor percentage (ER), progesterone receptor percentage (PR), and Ki67 expression level in PSMA expression subgroups in tumor blood vessels.

NEGATIVE PSMA EXPRESSION IN VESSELS						
	N	Mean	Median	Minimum	Maximum	SD
**ER%**	10	68.50	80.00	0.00	95.00	34.24
**PR%**	10	51.00	60.00	0.00	95.00	36.50
**Ki67**	10	16.90	17.50	2.00	45.00	12.46
**FOCAL PSMA EXPRESSION IN VESSELS**						
	**N**	**Mean**	**Median**	**Minimum**	**Maximum**	**SD**
**ER%**	27	60.74	80.00	0.00	95.00	40.61
**PR%**	27	40.93	30.00	0.00	95.00	39.52
**Ki67**	27	36.74	35.00	2.00	90.00	20.85
**WEAKLY POSITIVE PSMA EXPRESSION IN VESSELS**						
	**N**	**Mean**	**Median**	**Minimum**	**Maximum**	**SD**
**ER%**	19	68.95	80.00	0.00	95.00	34.54
**PR%**	19	42.63	40.00	0.00	95.00	35.33
**Ki67**	19	43.00	35.00	2.00	90.00	21.34
**STRONGLY POSITIVE PSMA EXPRESSION IN VESSELS**						
	**N**	**Mean**	**Median**	**Minimum**	**Maximum**	**SD**
**ER%**	42	35.95	10.00	0.00	95.00	41.01
**PR%**	42	17.05	0.00	0.00	90.00	30.96
**Ki67**	42	51.46	50.00	15.00	90.00	23.25

**Table 3:** Distribution of estrogen receptor percentage (ER), progesterone receptor percentage (PR), and Ki67 expression level in PSMA expression subgroups in tumor cells.

NEGATIVE PSMA EXPRESSION IN TUMOR CELLS						
	N	Mean	Median	Minimum	Maximum	SD
**ER%**	23	47.39	70.00	0.00	95.00	42.72
**PR%**	23	28.30	10.00	0.00	95.00	36.61
**Ki67**	23	35.04	30.00	2.00	85.00	26.99
**FOCAL PSMA EXPRESSION IN TUMOR CELLS**						
	**N**	**Mean**	**Median**	**Minimum**	**Maximum**	**SD**
**ER%**	59	58.14	80.00	0.00	95.00	39.25
**PR%**	59	34.05	10.00	0.00	95.00	37.19
**Ki67**	59	42.28	40.00	2.00	90.00	21.40
**WEAKLY POSITIVE PSMA EXPRESSION IN TUMOR CELLS**						
	**N**	**Mean**	**Median**	**Minimum**	**Maximum**	**SD**
**ER%**	10	45.00	45.00	0.00	95.00	45.70
**PR%**	10	36.10	20.50	0.00	90.00	40.51
**Ki67**	10	52.00	47.50	20.00	90.00	24.29
**STRONGLY POSITIVE PSMA EXPRESSION IN TUMOR CELLS**						
	**N**	**Mean**	**Median**	**Minimum**	**Maximum**	**SD**
**ER%**	4	0.00	0.00	0.00	0.00	0.00
**PR%**	4	0.00	0.00	0.00	0.00	0.00
**Ki67**	4	61.25	65.00	30.00	85.00	25.94

## Data Availability

The original contributions presented in the study are included in the article.

## References

[1] Giaquinto A.N., Sung H., Miller K.D., Kramer J.L., Newman L.A., Minihan A., Jemal A., Siegel R.L. (2022). Breast Cancer Statistics, 2022. CA Cancer J. Clin..

[2] Cardoso F., Kyriakides S., Ohno S., Penault-Llorca F., Poortmans P., Rubio I.T., Zackrisson S., Senkus E., ESMO Guidelines Committee (2019). Early breast cancer: ESMO Clinical Practice Guidelines for diagnosis, treatment and follow-up†. Ann. Oncol..

[3] Ceci F., Oprea-Lager D.E., Emmett L., Adam J.A., Bomanji J., Czernin J., Eiber M., Haberkorn U., Hofman M.S., Hope T.A. (2021). E-PSMA: The EANM standardized reporting guidelines v1.0 for PSMA-PET. Eur. J. Nucl. Med. Mol. Imaging.

[4] Denmeade S., Schwab M. (2016). Prostate-Specific Membrane Antigen. Encyclopedia of Cancer.

[5] O’Keefe D.S., Bacich D.J., Huang S.S., Heston W.D.W. (2018). A Perspective on the Evolving Story of PSMA Biology, PSMA-Based Imaging, and Endoradiotherapeutic Strategies. J. Nucl. Med. Off. Publ. Soc. Nucl. Med..

[6] Chang S.S., Reuter V.E., Heston W.D., Bander N.H., Grauer L.S., Gaudin P.B. (1999). Five different anti-prostate-specific membrane antigen (PSMA) antibodies confirm PSMA expression in tumor-associated neovasculature. Cancer Res..

[7] Grant C.L., Caromile L.A., Ho V., Durrani K., Rahman M.M., Claffey K.P., Fong G.H., Shapiro L.H. (2012). Prostate specific membrane antigen (PSMA) regulates angiogenesis independently of VEGF during ocular neovascularization. PLoS ONE.

[8] De Galiza Barbosa F., Queiroz M.A., Nunes R.F., Costa L.B., Zaniboni E.C., Marin J.F.G., Cerri G.G., Buchpiguel C.A. (2020). Nonprostatic diseases on PSMA PET imaging: A spectrum of benign and malignant findings. Cancer Imaging.

[9] Conway R.E., Rojas C., Alt J., Nováková Z., Richardson S.M., Rodrick T.C., Fuentes J.L., Richardson N.H., Attalla J., Stewart S. (2016). Prostate-specific membrane antigen (PSMA)-mediated laminin proteolysis generates a pro-angiogenic peptide. Angiogenesis.

[10] Unger C., Bronsert P., Michalski K., Bicker A., Juhasz-Böss I. (2022). Expression of Prostate Specific Membrane Antigen (PSMA) in Breast Cancer. Geburtshilfe Frauenheilkd.

[11] Tolkach Y., Gevensleben H., Bundschuh R. (2018). Prostate-specific membrane antigen in breast cancer: A comprehensive evaluation of expression and a case report of radionuclide therapy. Breast Cancer Res. Treat..

[12] Ross J.S., Schenkein D., Webb I. (2004). Expression of prostate specific membrane antigen in the neo-vasculature of non-prostate cancers. J. Clin. Oncol..

[13] Wernicke A.G., Varma S., Greenwood E.A., Christos P.J., Chao K.S.C., Liu H. (2014). Prostate-specific membrane antigen expression in tumor-associated vasculature of breast cancers. APMIS Acta Pathol. Microbiol. Immunol. Scand..

[14] Kasoha M., Unger C., Solomayer E.F., Bohle R.M., Zaharia C., Khreich F., Wagenpfeil S., Juhasz-Böss I. (2017). Prostate-specific membrane antigen (PSMA) expression in breast cancer and its metastases. Clin. Exp. Metastasis.

[15] Sartor O., de Bono J., Chi K.N., Fizazi K., Herrmann K., Rahbar K., Tagawa S.T., Nordquist L.T., Vaishampayan N., El-Haddad G. (2021). Lutetium-177-PSMA-617 for Metastatic Castration-Resistant Prostate Cancer. N. Engl. J. Med..

[16] Evans J.C., Malhotra M., Cryan J.F., O’Driscoll C.M. (2016). The therapeutic and diagnostic potential of the prostate specific membrane antigen/glutamate carboxypeptidase II (PSMA/GCPII) in cancer and neurological disease. Br. J. Pharmacol..

[17] Rahbar K., Ahmadzadehfar H., Kratochwil C., Haberkorn U., Schäfers M., Essler M., Baum R.P., Kulkarni H.R., Schmidt M., Drzezga A. (2017). German Multicenter Study Investigating 177Lu-PSMA-617 Radioligand Therapy in Advanced Prostate Cancer Patients. J. Nucl. Med. Off. Publ. Soc. Nucl. Med..

[18] Almuradova E., Seyyar M., Arak H., Tamer F., Kefeli U., Koca S., Sen E., Telli T.A., Karatas F., Gokmen I. (2024). The real-world outcomes of Lutetium-177 PSMA-617 radioligand therapy in metastatic castration-resistant prostate cancer: Turkish Oncology Group multicenter study. Int. J. Cancer.

[19] Kunikowska J., Czepczyński R., Pawlak D., Koziara H., Pełka K., Królicki L. (2022). Expression of glutamate carboxypeptidase II in the glial tumor recurrence evaluated in vivo using radionuclide imaging. Sci. Rep..

[20] Bertagna F., Albano D., Cerudelli E., Gazzilli M., Tomasini D., Bonù M., Giubbini R., Treglia G. (2020). Radiolabelled PSMA PET/CT in breast cancer. A systematic review. Nucl. Med. Rev. Cent. East. Eur..

[21] Sathekge M., Lengana T., Modiselle M., Vorster M., Zeevaart J., Maes A., Ebenhan T., Van de Wiele C. (2017). ^68^Ga-PSMA-HBED-CC PET imaging in breast carcinoma patients. Eur. J. Nucl. Med. Mol. Imaging.

[22] Turashvili G., Brogi E. (2017). Tumor Heterogeneity in Breast Cancer. Front. Med..

[23] Lu B., Natarajan E., Balaji Raghavendran H.R., Markandan U.D. (2023). Molecular Classification, Treatment, and Genetic Biomarkers in Triple-Negative Breast Cancer: A Review. Technol. Cancer Res. Treat..

[24] Ghosh A., Heston W.D.W. (2004). Tumor target prostate specific membrane antigen (PSMA) and its regulation in prostate cancer. J. Cell Biochem..

[25] Morgenroth A., Tinkir E., Vogg A.T.J., Sankaranarayanan R.A., Baazaoui F., Mottaghy F.M. (2019). Targeting of prostate-specific membrane antigen for radio-ligand therapy of triple-negative breast cancer. Breast Cancer Res..

[26] Nguyen D.P., Xiong P.L., Liu H., Pan S., Leconet W., Navarro V., Guo M., Moy J., Kim S., Ramirez-Fort M.K. (2016). Induction of PSMA and internalisation of an anti-PSMA mAb in the vascular compartment. Mol. Cancer Res..

[27] Eppard E., de la Fuente A., Benešová M., Khawar A., Bundschuh R.A., Gärtner F.C., Kreppel B., Kopka K., Essler M., Rösch F. (2017). Clinical translation and first in-human use of [^44^Sc]Sc-PSMA-617 for PET imaging of metastasized castrate-resistant prostate cancer. Theranostics.

[28] Ruigrok E.A.M., van Vliet N., Dalm S.U., de Blois E., van Gent D.C., Haeck J., de Ridder C., Stuurman D., Konijnenberg M.W., van Weerden W.M. (2021). Extensive preclinical evaluation of lutetium-177-labeled PSMA-specific tracers for prostate cancer radionuclide therapy. Eur. J. Nucl. Med. Mol. Imaging.

[29] Andryszak N., Świniuch D., Wójcik E., Ramlau R., Ruchała M., Czepczyński R. (2024). Head-to-Head Comparison of [^18^F]PSMA-1007 and [^18^F]FDG PET/CT in Patients with Triple-Negative Breast Cancer. Cancers.

